# A Portable Waist-Loaded Soft Exosuit for Hip Flexion Assistance with Running

**DOI:** 10.3390/mi13020157

**Published:** 2022-01-21

**Authors:** Lingxing Chen, Chunjie Chen, Xin Ye, Zhuo Wang, Yao Liu, Wujing Cao, Shaocong Chen, Xinyu Wu

**Affiliations:** 1 Shenzhen Institute of Adanced Technology, Chinese Academy of Sciences, Shenzhen 518055, China; lx.chen@siat.ac.cn (L.C.); xin.ye@siat.ac.cn (X.Y.); zhuo.wang@siat.ac.cn (Z.W.); ll.liu1@siat.ac.cn (Y.L.); wj.cao@siat.ac.cn (W.C.); sc.chen1@siat.ac.cn (S.C.); xy.wu@siat.ac.cn (X.W.); 2 Shenzhen College of Advanced Technology, University of Chinese Academy of Sciences, Shenzhen 518055, China; 3 Guangdong Provincial Key Lab of Robotics and Intelligent System, Shenzhen Institute of Advanced Technology, Chinese Academy of Sciences, Shenzhen 518055, China; 4 Guangdong-Hong Kong-Macao Joint Laboratory of Human-Machine Intelligence-Synergy Systems, Shenzhen Institute of Advanced Technology, Chinese Academy of Sciences, Shenzhen 518055, China

**Keywords:** waist-loaded soft exosuit, running, hip flexion, motion flexibility, metabolic rate

## Abstract

The soft exosuit is an emerging robotics, which has been proven to considerably reduce the metabolic consumption of human walking and running. However, compared to walking, relatively few soft exosuits have been studied for running. Many soft exosuits used for running are worn on the back and with a heavy weight load, which may cause instability while running and potentially increase metabolic consumption. Therefore, reducing the weight of the whole soft exosuit system as much as possible and keeping the soft exosuit close to the center of gravity, may improve running stability and further reduce metabolic consumption. In this paper, a portable waist-loaded soft exosuit, the weight of which is almost entirely concentrated at the waist, is shown to assist hip flexion during running, and justifies choosing to assist hip flexion while running. As indicated by the experiments of motion flexibility, wearing the waist-loaded soft exosuit can assist in performing many common and complex motions. The metabolic consumption experiments proved that the portable waist-loaded soft exosuit reduces the metabolic consumption rate of wearers when jogging on the treadmill at 6 km per hour by 7.79% compared with locomotion without the exosuit. Additionally, at the running speed of 8 km per hour, using the waist-loaded soft exosuit can reduce metabolic consumption rate by 4.74%. Similarly, at the running speed of 10 km per hour, it also can be reduced by 6.12%. It is demonstrated that assisting hip flexion for running is also a reasonable method, and wearing the waist-loaded soft exosuit can keep human motion flexibility and reduce metabolic consumption.

## 1. Introduction

In recent years, lower limb exoskeletons have been widely used to restore walking ability for patients with lower limb motor dysfunction, or to augment locomotion ability of healthy people [1]. The research and development of exoskeleton robots are of great significance to strengthen medical security, improve personnel health, and enhance the work efficiency of personnel in special fields [2]. Generally speaking, lower limb exoskeletons are divided into two categories according to different principles and materials [3,4]: rigid exoskeleton and soft exosuit, whose main difference lies in the use of materials. Rigid exoskeletons are connected by rigid components, while soft exosuits are mostly composed of textile compositions [5]. From the point of view of ordinary wearers, soft exosuit has better shape adaptability, lower-key appearance, better compliance, and much lighter weight than traditional exoskeleton, making it more suitable to wear [6,7].

The wearable soft exosuit is an emerging robot with appreciable wearability [8], and the joint torque generated is also sufficient to enhance the user’s movement and physical capabilities. As for the soft exosuit that assists with walking, the Wyss Institute for Biologically Inspired Engineering laboratory developed a Bowden cable-based multi-joint lower limb soft exosuit prototype with DARPA funding [9]. The prototype, of which the total mass is 10.1 kg, uses flexible fabric to replace the traditional rigid structure and utilizes the synergistic enhancement effect of lower limb joints, and can reduce the wearer by an average of 6.4 ± 3.9% [5]. The Herr team of the Massachusetts Institute of Technology Media Lab (Media Lab) demonstrated an ankle joint booster exoskeleton. The total mass of the system is 3.85 kg, which can reduce the wearer’s metabolic value by 11 ± 4% during walking [9]. The Biorobotics Lab showed an exoskeleton system that can achieve ankle joint plantar flexion motion assistance and the system is equipped with a plantar pressure sensor, which can reduce the gastrocnemius muscle activity of the wearer by 3.4% [10]. Juanjuan Zhang et al. proposed a human-in-the-loop (HIL) optimization method for the assistance torque generated by an ankle exoskeleton to minimize human metabolic consumption during walking by customized control parameters (including assistance time and force peak, etc.) for users [11]. In order to further optimize the control performance, Ye Ding et al. used a Bayesian optimization method on the basis of HIL to continuously optimize the control parameters suitable for users during walking [12].

The above soft exosuits are all used to assist users during walking. However, running is also a very common human activity, so it is important and reasonable to research the devices that assist human running. Jinsoo Kim et al. showed that a portable exosuit carried on the back that assists hip extension can reduce the metabolic rate of treadmill walking at 1.5 m per second by 9.3% and that of running at 2.5 m per second by 4.0%, compared with locomotion without the exosuit [13]. Jaeha Yang et al. proposed a passive exosuit (without power) which was fabricated using textile materials and elastic bands to assist hip flexion that can reduce the user’s metabolism by 4% at a running speed of 2.5 m/s [14].

A single joint assisted lower limb soft exosuit can be divided into hip joint assistance, knee joint assistance, and ankle joint assistance [15,16,17]. The soft exosuit’s assistance of the hip joint can be divided into two categories: assisting hip flexion and assisting hip extension. In this paper, we propose a novel waist-loaded soft exosuit that is used to assist hip flexion during running. There are some reasons for choosing this method of assistance during running, such as walking or running, is coordinated by the musculoskeletal system. In the walking gait, the plantar flexion movement consumes the most energy, followed by the hip extension movement, while the hip flexion movement consumes little energy, because the thigh obtains enough energy from plantar-flexion movement in the pre-swing phase, which can meet the swinging limb movement in the swinging phase during walking [18]. Therefore, it is the most economical to assist plantar flexion or hip extension during walking at low speeds. However, when the human body’s movement speed gradually increases from walking gait to running gait, all the energy used for plantar flexion movement is transferred to the body’s center of gravity and used to push the human body forward. At the same time, the lower limbs need to actively contract the hip flexors, such as psoas muscle and iliopsoas muscle, in order to complete the swing phase. With the increase in running speed, the lower limbs need to complete the swing phase at a faster speed [19,20], that is, the hip flexors such as psoas muscle and iliopsoas muscle need to actively output more energy [21]. Therefore, during running, it is necessary to assist the hip flexion exercise to reduce muscle fatigue of the hip flexor group and improve the body’s exercise capacity. In addition, as the running speed increases, the time of the hip joint flexion phase gradually increases or even exceeds the time of the hip joint extension. This data proof will be given in Section 2.

We have developed several soft exosuits [1,22,23,24], including walking assistance and weight transfer. However, it is also important that the soft exosuit was used to assist running, and it can be used for normal running or even marathons in future works. In this paper, we present a novel waist-loaded soft exosuit of which weight is almost entirely concentrated around the waist (near the body’s center of gravity) to assist hip flexion for running, and the major contributions of this work are as follows:

(1) A portable waist-loaded soft exosuit is proposed to assist hip joint flexion during running, and the reasons for choosing to assist hip joint flexion during running are explained;
(2) The waist-loaded soft exosuit focuses almost all of its weight at the waist (near the center of gravity), which makes it a better fit for the human body and improves running stability;
(3) The motion flexibility of the waist-loaded soft exosuit is tested, and it is also proved that the use of the waist-loaded soft exosuit could reduce metabolic consumption during running.

In this paper, a novel waist-loaded soft exosuit is proposed to assist hip joint flexion during running. The prototype of soft exosuit will be introduced in Section 2, which includes structure design, and control method. Then, Section 3 will illustrate how to evaluate the performance of the waist-loaded soft exosuit through the motion flexibility and the metabolic consumption experiments. The discussion between the waist-loaded soft exosuit and other soft exosuits will show in Section 4. Finally, the conclusion will be presented in Section 5.

## 2. Waist-Loaded Soft Exosuit Design

The soft exosuit is an emerging wearable robot that can help the elderly and patients with rehabilitation training and reducing metabolic consumption [22,25]. In this section, the design concept, system overview, assistance force control and the control strategy of the waist-loaded soft exosuit will be in described detail.

### 2.1. Inspired Design Concept

Section 1 has explained that it is necessary to provide users hip flexion assistance with running, which can reduce muscle fatigue for the hip flexion muscle group and enhance human movement ability. When exercising, core strength (the core muscle group mainly concentrated in the waist, abdomen, and buttocks) is the most important, because the body’s center of gravity is located in the waist. Therefore, exercising these muscles can help people better overcome external interference and maintain balance. Similarly, placing weights on the waist is more stable than placing weights on other parts of the body, including the back, shoulders, and legs [26,27].

**Figure 1:**
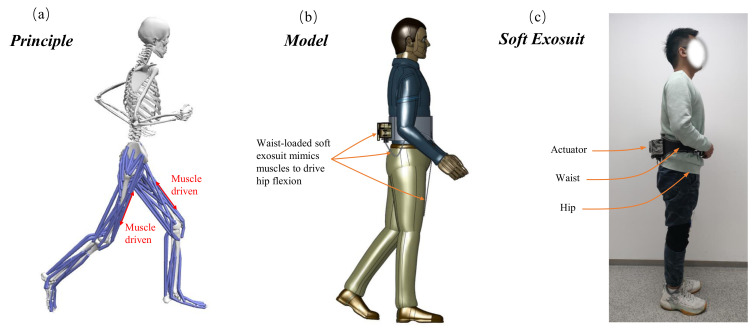
The design concept of waist-loaded soft exosuit. (**a**) Working principle of waist-loaded soft exosuit during running. (**b**) 3D model of the waist-loaded soft exosuit to show the function simulation. (**c**) Prototype of the waist-loaded soft exosuit.

Inspired by the human body’s center of gravity, the weight of waist-loaded soft exosuit is almost worn on the waist of users in the form of a belt, which can make people run more smoothly. Especially in running scenarios, the body’s center of gravity is shifted up and down. Therefore, it is reasonable and stable to wear the soft exosuit in the form of a belt on the waist compared to wearing it on the rest of the body. As shown in Figure 1, the waist-loaded soft exosuit is worn near the center of gravity and provides assistance for hip flexion during running.

### 2.2. System Overview

The soft exosuit is a wearable walking assistance device, which can provide assistance for hip flexion [28], help the elderly and patients with rehabilitation training, and reduce metabolic consumption [29]. In this research, we design a novel waist-loaded soft exosuit whose weight is almost concentrated on the waist (near the center of gravity). The waist-loaded soft exosuit can assist the wearers to perform high-frequency flexion of the hip joint during running, and use Bowden cables to transmit moment consistent with the combined moment produced by different biological muscles.

**Figure 2:**
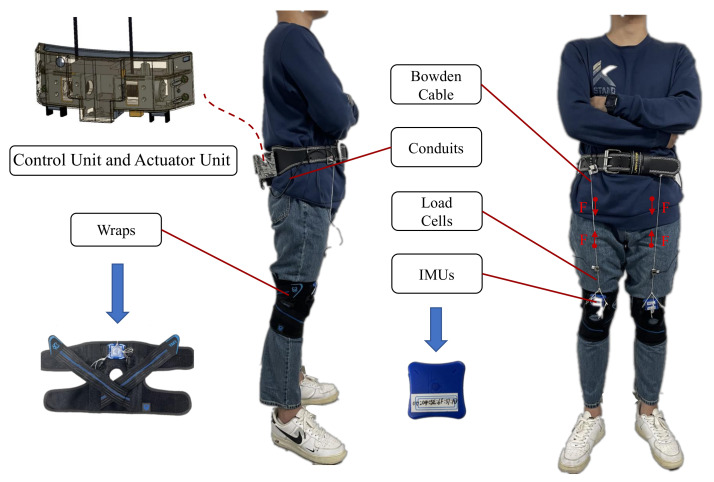
The waist-loaded soft exosuit. The whole system is almost worn on the waist of humans. The F represents the direction of assistance force.

As shown in Figure 2, the waist-loaded soft exosuit designed in this paper is mainly composed of control unit, actuator unit, Bowden cables, Inertial Measurement Units (IMUs), load cells, flexible wraps, and conduits. The control unit, which is made up of an ST 32-bit microcontroller (MCU, stm32F427IIH, ST Inc, The United States) and Bluetooth modules, is used to communicate with the motor controller and sensors. A 32-bit low-power microprocessor unit (stm32L431c8t, Microchip ST Inc, The United States) is used to read analog force signals from the load cell and read motion information from the IMU via a universal asynchronous receiver/transmitter (UART). The actuator unit consists of motors (M2006, DJI, Shenzhen, China), motor speed controllers (C610, DJI, Shenzhen, China) and a reel (radius is 10 mm). The actuator unit, control unit, and battery (2900 mAh, can power the waist-loaded soft exosuit for about two hours) are integrated on the waist (near the wearer’s center of gravity) through the waist belt to reduce running instability caused by the distal mass. The whole system has a compact structure and is made of lightweight aluminum alloy and resin materials. The function of assisting hip joint flexion is completed by two high-power low-inertia motors (M2006, DJI, Shenzhen, China) of the actuator unit, each of which is connected to a 36:1 ratio gearbox to drive a reel (radius is 10 mm). IMUs (including gyroscopes and accelerometers) which are placed on the flexible wraps, are used to detect gait events, and are important sensors for human gait information collection [30]. One end of the Bowden cable is connected with the outlet of the actuator unit, and the other end is connected and fixed on the flexible wraps. The assistance force generated by the drive motor is transmitted by Bowden cable contraction to the flexible wraps to assist hip flexion. We use a load cell (ZNLBS-v1, Chino Sensor, China) to measure the hip flexion assistance force of each leg, and it places between the Bowden cable and the thigh flexible wraps. The total mass of the entire waist-loaded soft exosuit system is 1.932 kg. The weight distribution of the waist-loaded soft exosuit is shown in Table 1.

### 2.3. Hip Flexion Assistance Force Control

Different joints produce different biological joint moments during walking and running [31]. Hip flexion is an important part of hip motion. In this paper, a waist-loaded soft exosuit is proposed for hip flexion assistance with running. In this section, the biological hip moment changes at different running speeds will be analyzed. Simultaneously, the biological hip moment curve is used as the theoretical reference of the hip flexion assistance moment, and we will further explain the reasons for using the hip flexion assistance in running scenarios according to the biological hip moment data at different running speeds.

**Figure 3:**
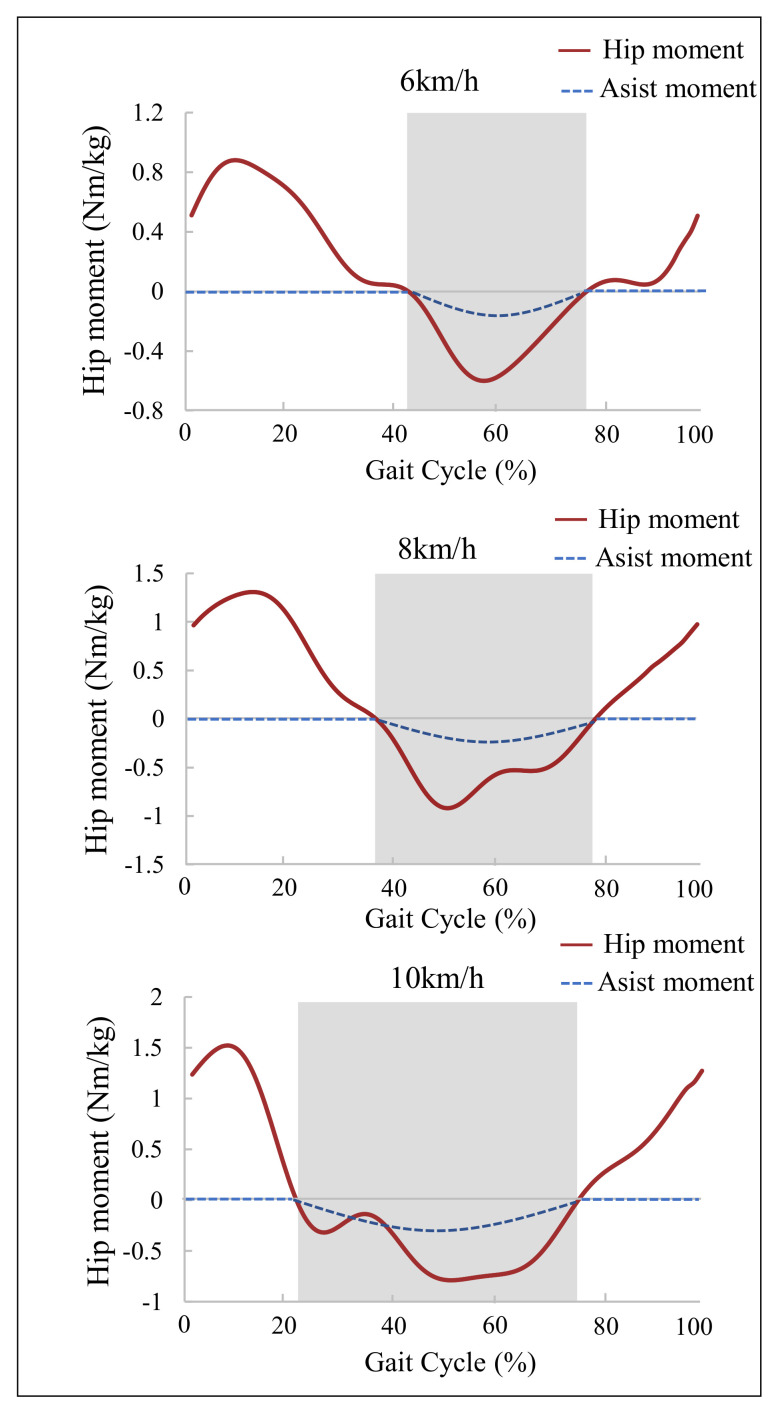
Biological hip moment and assistance moment under the running speed of 6 km/h, 8 km/h, and 10 km/h, respectively. The biological hip moment is the average moment of all subjects. Assistance moment is the desired moment of soft exosuit which is determined by the biological hip moment. The shaded part represents the hip flexion.

For analyzing the change of biological hip moment at different running speeds, five healthy male subjects (25 ± 2 years old; 74.6 ± 9.6 kg weight; 177 ± 4 cm height) are invited to do the motion capture experiment by Vicon (Oxford metrics Limited, England). Each subject runs on the Tandem Treadmill (AMTI Force-Sensing Treadmills, America) at a speed of 6 km/h, 8 km/h and 10 km/h, respectively (6 km/h, 8 km/h and 10 km/h are jogging speed, moderate running speed and fast running speed, respectively). Firstly, the biological hip moment data of five subjects is collected, then smoothed and filtered through MATLAB (MathWorks, Natick, MA, USA). Finally, the subjects’ biological hip moment data at the same running speed are averaged, as shown in Figure 3. In order to verify the credibility of the motion capture experiment, the biological hip joint moment curve obtained from the motion capture experiment is compared with the data of Kim et al. [13] (running motion data from seven participants running at 2.5 m/s, provided by Kim et al. as Supplementary Material [13], were used for the analysis). It can be found that both the biological hip moment curve have similar trends during running, which proves the validity of the biological hip joint data in this paper. As shown in Figure 3, it can be discovered that the peak and phase of the biological hip moment are inconsistent at different running speeds. Especially during the hip flexion phase (the gray shaded area in Figure 3), the duration of hip flexion gradually increases and even exceeds the duration of hip extension as running speed increases. Therefore, it is reasonable and necessary to use waist-loaded soft exosuit to assist hip flexion with running.

The control strategy of the soft exosuit is mainly to change the amplitude and phase of the force [32]. As shown in Figure 3, the peak moment of the hip joint and the assistance phase are inconsistent at different running speeds. Because the biological hip joint moment is the theoretical basis of the waist-loaded soft exosuit assistance moment, therefore different assistance moments need to be set up for different speeds. In order to prevent excessive assistance moments from damaging subjects, refer to [13], the sine curve is used to replace the hip joint moment, and the curve obtained after 12% scaling is the final assistance moment curve which is shown in Figure 3. Therefore, the peak assistance moments of waist-loaded soft exosuit are 0.072 Nm/kg, 0.096 Nm/kg, and 0.108 Nm/kg at running speeds of 6 km/h, 8 km/h, and 10 km/h, respectively. The zero point of the moment curve is determined by the gait event, that is, the assist start time and end time can be determined by the angle threshold during running.

### 2.4. Control Strategy

**Figure 4:**
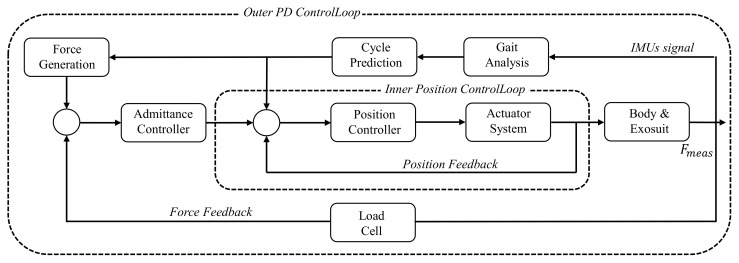
The waist-loaded soft exosuit control system.

Cao et al. have proposed a hierarchical control strategy based on iterative learning (POILC) [22], and we have proposed a simplified hierarchical control strategy [31] in previous works, which can reduce the control complexity of the POILC. In this work, a hierarchical control strategy with gait cycle prediction is introduced. The control diagram is shown in Figure 4, and admittance control has shown good performance in single motion assistance of soft hip exoskeleton [33]. In this paper, a proportional-derivative (PD) controller is utilized as admittance controller, which can be expressed by Equation (1)

(1) $$u_{t}=K_{p}e_{t}+K_{d}\Delta e_{t}$$

where $K_{p}$ϵ$R^{2\times 2}$ and $K_{d}$ϵ$R^{2\times 2}$ denote, respectively, the coefficient matrices of the proportional and derivative controllers [34].

IMUs send the collected human gait information (Euler angle and angular velocity) to the microcomputer (STM32F427IIH, ARM Cortex-M3) via Bluetooth, then the microcomputer stores the data of five consecutive gait cycles. Finally, the weight coefficient vector is used to predict the next gait cycle after weighted average calculation. The weight coefficients of these five consecutive gait cycles are assigned according to the distance from the current time. The closer to the current time, the bigger the weight coefficient. The specific coefficient is shown in the Equation (2)

(2) $$\left[0.7,0.2,0.05,0.03,0.02\right]$$

It is reasonable to utilize the distance from the current time to assign the weight coefficient, because human movement is continuous, when a person is walking normally, the change of speed and step length is gradual (of course, unexpected events are unpredictable). For the value in reinforcement learning (RL), there is a discount coefficient to reduce the impact of future rewards on current actions. The whole control system includes Inner Position ControlLoop and Outer PD ControlLoop. The Inner Position ControlLoop is used for the position feedback of the motor system. The Outer PD ControlLoop regards the PD controller as the main controller, and calculates the feedback data of the load cell to make the whole control system more stable.

## 3. Evaluation of the Soft Exosuit

In this section, we discuss the experiment of the waist-loaded soft exosuit. Because the soft exosuit needs to assist human movement without interfering with normal human motion, its flexibility needs to be analyzed. Simultaneously, the gas analysis equipment (MasterScreen PFT System, Gerge, Germany) is utilized to evaluate the performance of waist-loaded soft exosuit.

### 3.1. Experimental Setup and Protocol

Five male subjects are invited to participate in the experiment, whose specific physical conditions are shown in the Table 2. The experiment was approved by the Medical Ethics Committee of Shenzhen Institute of Advanced Technology ((SIAT)-IRB-200715-H0512 (valid time from January 2020 to December 2022)), and the content of the experiment and its possible impact were explained to all the participants before conducting the experiment.

### 3.2. Motion Flexibility

The motion flexibility of the users wearing the soft exosuit can show the wearable performance of the waist-loaded soft exosuit. Because the soft exosuit needs to provide assistance force without affecting the user’s normal motion. If the users wear the waist-loaded soft exosuit to complete some common daily motion, which can indicate that it will not interfere with the human motion.

**Figure 5:**
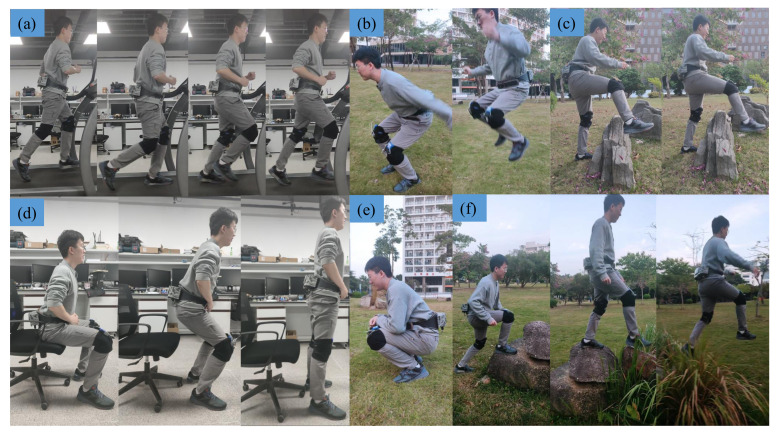
The motion flexibility of the users wearing the waist-loaded soft exosuit: (**a**) running, (**b**) jumping, (**c**) across obstacle, (**d**) sit down, (**e**) crouch, and (**f**) traverse complex terrain.

We first invite a subject to wear the waist-loaded soft exosuit to complete some scenes of running and special motion. As shown in Figure 5, the six groups of motions include running, jumping, across obstacle, sit down, crouch, and traverse complex terrain (all the above motions are completed when the waist-loaded soft exosuit provides assistance force for users). It can be found that the waist-loaded soft exosuit worn by the subjects can easily complete some common motions and will not cause obstacles to the human body, which proves that the waist-loaded soft exosuit has strong motion flexibility and human body adaptability.

### 3.3. Metabolic Consumption Experiment

**Figure 6:**
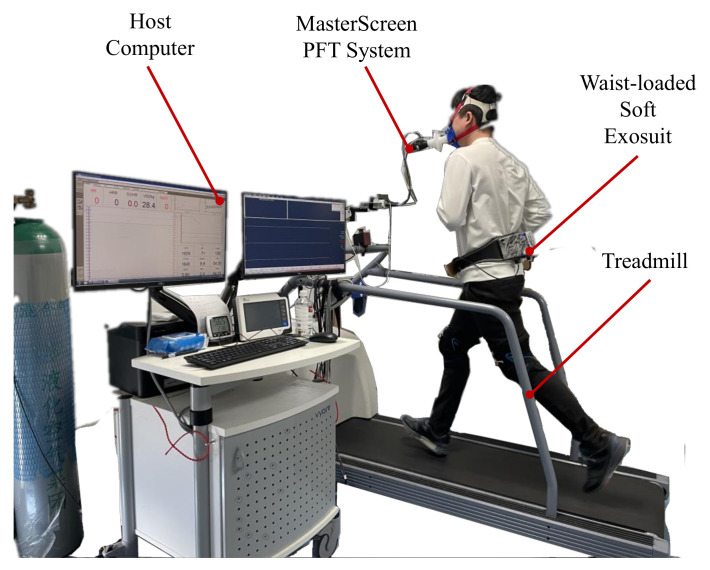
Evaluation experiment of the waist-loaded soft exosuit with MasterScreen PFT System. Subjects wear the waist-loaded soft exosuit and measure metabolic consumption.

The final study is intended to evaluate the user’s metabolic consumption performance of running with the assistance of the proposed waist-loaded soft exosuit. As shown in Figure 6, the experimental devices are made up of the waist-loaded soft exosuit, a host computer, the MasterScreen PFT System, and a treadmill. The subject runs on a treadmill (the treadmill speeds set 6 km/h, 8 km/h, and 10 km/h) with the waist-loaded soft exosuit. At the same time, the MasterScreen PFT System is utilized to obtain the user’s metabolic consumption data and do the evaluation experiments of waist-loaded soft exosuit.

The MasterScreen PFT System calculates human metabolic consumption (*W*) by using the volume of carbon dioxide exhaled and oxygen inhaled. Metabolic consumption experiment is also the most commonly used evaluation method for wearable robots, so we utilize the MasterScreen PFT System to evaluate the assistance performance of the waist-loaded soft exosuit. Five healthy male subjects (25 ± 2 years old; 74.6 ± 9.6 kg weight; 177 ± 4 cm height), as shown in the Table 2, are recruited to participate in this metabolic consumption experiments. The entire metabolic consumption experiment needs to be performed at three different running speeds. Therefore, each subject needs to complete without using the exosuit, wearing the exosuit without power, and wearing the exosuit with power, respectively, at the speed of 6 km/h, 8 km/h, and 10 km/h. That is, each subject has to complete 9 metabolic consumption tests. Each test lasts 15 min and has a 15 min rest time after the end, to ensure the validity of metabolic consumption data.

The metabolic is computed through the rate of Oxygen consumption (VO2) and the Carbon dioxide emission (VCO2) according to Brockway equation [35]:

(3) $$E_{Metabolic}\left(W\right)=\left(c_{1}VO_{{}_{2}}+c_{2}VCO_{{}_{2}}\right)/60$$

where the $E_{Metabolic}$ is the energy consumption rate, coefficients $c_{1}$ and $c_{2}$ are 16.68 and 4.51, respectively, and $VO_{2}$ and $VCO_{2}$ are the volumes of oxygen in and carbon dioxide out which are measured through MasterScreen PFT System.

**Figure 7:**
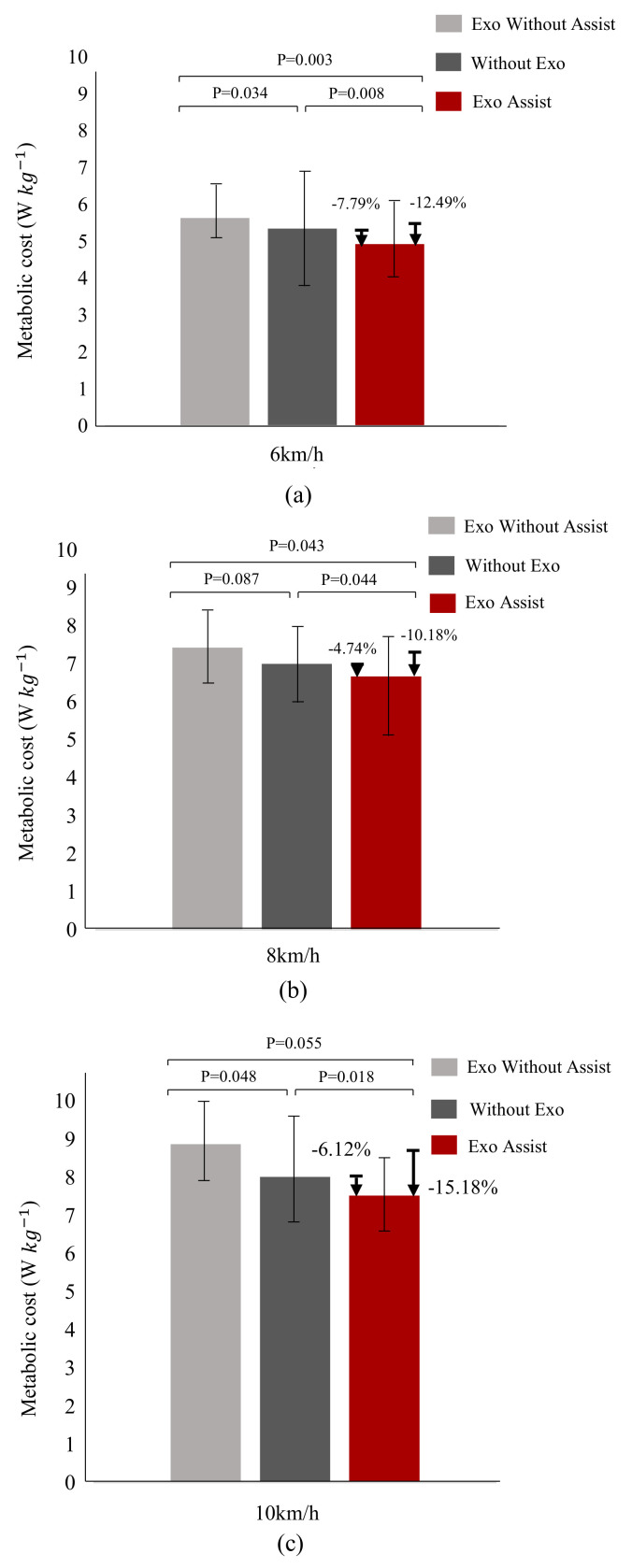
Metabolic consumption, which is obtained by using a MasterScreen PFT System. (**a**) In 6 km/h jogging speed, using the waist-loaded soft exosuit to do metabolic consumption experiments through wearing the exosuit without power, without using the exosuit and wearing the exosuit with power, respectively. (**b**) Similar to (**a**), in 8 km/h running speed, using the waist-loaded soft exosuit to do metabolic consumption experiments. (**c**) Similar to (**a**), in 10 km/h running speed, using the waist-loaded soft exosuit to do metabolic consumption experiments.

As shown in Figure 7, Exo Without Assist, Without Exo, and Exo Assist represent wearing the exosuit without power, without using the exosuit, and wearing the exosuit with power, respectively. As shown in the Figure 7a, under the 6 km/h jogging experiment, it can be discovered that compared with wearing the exosuit without power, the metabolic consumption of using the waist-loaded soft exosuit is reduced by about 12.06%, and compared with without using the exosuit, it also can be reduced by 7.79%. From the Figure 7b, under the 8 km/h running experiment, it also can be found that compared with wearing the exosuit without power, the metabolic consumption of using the waist-loaded soft exosuit is reduced by about 10.18%, and compared with without using the exosuit, it also can be reduced by 4.74%. Similarly, under the 10 km/h running experiment, it can be discovered that compared with wearing the exosuit without power and without using the exosuit, the metabolic consumption of using the waist-loaded is reduced by about 15.18% and 6.12%, respectively.

As can be seen from Figure 7, with the increase in running speed (from 6 km/h to 10 km/h), the overall metabolic consumption (W/kg) will also increase. This is reasonable because the increase in running speed means that the human body needs to provide more energy to drive muscles contraction. In the three running speed experiments, the metabolic consumption of wearing the exosuit without power (in this case, the waist-loaded soft exosuit is only considered as external load) exceeds that of wearing the exosuit with power and without using the exosuit. Simultaneously, it can be discovered that the metabolic consumption of wearing the exosuit with power is less than that in the other two groups of the same running speed experiments. Therefore, the metabolic consumption experiment proved that the waist-loaded soft exosuit to assist hip flexion can help users reduce metabolic consumption during. Simultaneously, it also demonstrates that assisting the human hip flexion during running is also a good solution.

## 4. Discussion

This work presents a portable waist-loaded soft exosuit of which almost all of the weight is concentrated near the body’s center of gravity, to assist hip flexion during running. Our team uses the Vicon and Tandem Treadmill to analyze the biological hip moment curve. The motion flexibility and net metabolic consumption rate are also evaluated. The detailed comparison of typical soft exoskeletons is presented in Table 3. Compared to [11,16,26,36,37], which are all lower limb soft exosuit to assist users during walking, the proposed portable waist-loaded soft exosuit can be used to assist hip flexion for running. One of the most obvious differences between [13] and this work is that [13] assisted hip extension and weighs up to 5.004 kg, while our waist-loaded soft exosuit assists hip flexion for running and weighs 1.932 kg, which has a lighter mass. The author claimed, in [37], that multi-joint assistance, including the assist torque of the ankle and hip joints, is beneficial to reducing the net metabolic rate, indicating that multi-joint assistance reduces net metabolic rate by 16.93% during walking compared with the absence of any external force assistance. Although the soft exoskeleton in [37] can more significantly reduce the net metabolic rate, but its weight is nearly twice that of our waist-loaded soft exosuit. If the soft exosuit only assists ankle joints are probably very light in weight, the ankle assistance in [26] is unpowered soft exoskeleton, of which the weight is only 0.816–1.006 kg, and can reduce the metabolic consumption rate by 7.2 ± 2.6%. Despite its light mass, the rise potential of an unpowered exoskeleton is not as good as that of a powered soft exosuit because of the law of conservation of energy, and our waist-loaded soft exosuit is to assist hip flexion during running. From the comparison between [11,13,16,26,36,37] and this work, it is found that running reduces metabolic value not as significantly as walking reduces metabolism. Overall, in running assistance applications, the waist-loaded soft exosuit, on the one hand, can reduce the metabolic consumption of the wearer for running, on the other hand, it also has a high degree of motion flexibility, allowing the wearer to complete many motions more freely.

### 4.1. Discussion of This Work

Walking, climbing, and long-distance running (marathon, etc.) are all very common exercise methods. Sometimes the energy consumed to reach the destination is beyond the limit of a person, and the use of the exoskeleton can help improve the person’s athletic ability. Therefore, for running, a portable waist-loaded soft exosuit is proposed to assist the human hip flexion during running, so as to reduce metabolic consumption and increase the user’s athletic ability. Most weight of the soft exosuit is concentrated on the waist to improve the stability of running. The reasons why we choose to assist hip flexion during running are explained in Section 1. When the human body’s movement speed gradually increases from walking gait to running gait, all the energy used for plantar flexion movement is transferred to the body’s center of gravity and used to push the human body forward. At the same time, the lower limbs need to actively contract the hip flexors, such as psoas muscle and iliopsoas muscle, to complete the swing phase. With the increase in running speed, the lower limbs need to complete the swing phase at a faster speed, that is, the hip flexors, such as psoas muscle and iliopsoas muscle, need to actively output more energy [21]. It was proven by the hip moment experiment in Section 2 that, especially during the hip flexion phase, the duration of hip flexion gradually increases and even exceeds the duration of hip extension as running speed increases. Therefore, it is reasonable to use waist-loaded soft exosuit to assist hip flexion with running.

### 4.2. Limitations of This Work

Despite this, the waist-loaded soft exosuit which assists hip flexion for running is novel, and significant motion flexibility and metabolic benefit is achieved. There are some limitations to this work. Firstly, compared with the reduced metabolism during walking in other papers, the reduction during running is still not enough. Secondly, the waist-loaded soft exosuit in the paper only passed the motion flexibility and metabolic test, without mechanical fatigue test, and does not test the duration of continuous operation of the whole system. Finally, to use waist-loaded exoskeleton for outdoor running (marathon, etc.), further optimization is needed, including the factors of overall weight and battery life.

## 5. Conclusions

This work presents a novel portable waist-loaded soft exosuit of which weight is almost concentrated on the waist, to assist hip flexion during running. For the reason why we choose to assist hip flexion during running are explained, because, with the increase in running speed, the lower limbs need to complete the swing phase at a faster speed, that is, the hip flexors, such as psoas muscle and iliopsoas muscle, need to actively output more energy. Simultaneously, during the hip flexion phase the duration of hip flexion gradually increases and even exceeds the duration of hip extension as running speed increases through hip moment experiment proving. Indicated from the experiments of motion flexibility, wearing the waist-loaded soft exosuit can perform many common and complex motions. The metabolic consumption experiments proved that the portable waist-loaded soft exosuit reduces the metabolic consumption rate of wearers when jogging on the treadmill at 6 km/h by 7.79% compared with locomotion without the exosuit. Additionally, at the running speed of 8 km/h, using the waist-loaded soft exosuit can reduce metabolic consumption rate by 4.74%. Similarly, at the running speed of 10 km/h, it also can be reduced by 6.12%.

In summary, this study focuses on assisting running, explaining the reason why to assist hip flexion. In the future, we can further optimize the waist-loaded soft exosuit and apply it to outdoor running, such as marathons and other sports.

## Figures and Tables

**Table 1:** The mass distribution of the waist-loaded soft exosuit.

Part	Mass (kg)	Location
Waist belt	0.32	Waist
Actuator	0.214	Waist
Battery	0.30	Waist
MCU	0.08	Waist
Conduits	0.052	Waist
IMUs	0.024	Thigh
Wraps	0.38	Thigh
Load cells	0.05	Thigh
Other component	0.512	Waist

**Table 2:** The physical conditions of subjects.

Subjects	Gender	Height (cm)	Weight (kg)	Age (Years Old)
LX	Male	176	72	25
XY	Male	173	75	25
ZW	Male	178	80	27
CS	Male	175	78	23
LL	Male	183	65	26

**Table 3:** Comparison of typical soft exoskeletons.

Research	Actuator Location	Assistance Scenario	Assistance Mode	Weight (kg)	Power	Metabolic Cost (%)
Zhang et al. [11]	Platform	Walking	Ankle plantar flexion	∖	Powerd	5.9
Kim et al. [13]	Wearer	Walking and Running	Hip extension	5.004	Powerd	9.3
Jim et al. [36]	Wearer	Walking	Hip flexion	∖	Powerd	5.9
Sangjun et al. [37]	Wearer	Walking	Hip extension and flexion and Ankle plantar flexion	5.1	Powerd	16.93
Ding et al. [16]	Platform	Walking	Hip extension and flexion and Ankle plantar flexion	∖	Powerd	14.6
Collins et al. [26]	Wearer	Walking	Ankle plantar flexion	0.816–1.006	Unpowered	7.2 ± 2.6
This work	Wearer	Running	Hip flexion	1.932	Powered	7.79

## Data Availability

The data that support the findings of this study are available on request from the corresponding author.

## Abbreviations

The following abbreviations are used in this manuscript:

HIL	Human-in-the-loop
IMU	Inertial measurement unit
STM32	STMicroelectronics 32-bit Series Microcontroller Chip
PD	Proportional-derivative
RL	Reinforcement learning
